# Public perception and acceptance of biotechnology in agriculture: insights from Poland

**DOI:** 10.3389/fbioe.2026.1983759

**Published:** 2026-10-22

**Authors:** Woźniak-Gientka Ewa, Tyczewska Agata

**Affiliations:** Institute of Bioorganic Chemistry, Polish Academy of Sciences, Poznań, Poland

**Keywords:** biotechnology, GMO, new genomic techniques, public opinion, social acceptance

## Abstract

Agricultural biotechnology encompasses a range of technologies, including genetically modified organisms (GMOs), which have been developed and applied in agriculture for several decades, as well as the more recently developed new genomic techniques (NGTs), which have attracted increasing attention in the context of challenges related to sustainable agriculture and food security. However, its successful implementation depends not only on technological progress but also on public acceptance. This study presents a structured literature review synthesizing the existing evidence on public acceptance of agricultural biotechnology in Poland. Relevant studies were identified through a structured search of the Scopus and Web of Science databases using predefined search terms related to public acceptance, perceptions, attitudes, GMOs, NGTs, and Poland. The reviewed literature was analyzed to identify patterns in public awareness, perceptions, attitudes, and the main determinants influencing technology acceptance. The review shows that research conducted in Poland has focused predominantly on GMOs, while empirical evidence on public perceptions of NGTs remains scarce. Public acceptance is strongly influenced by the application context, perceived risks and benefits, trust in science and institutions, and the level of public awareness. Biotechnology applications associated with health and environmental benefits generally receive greater support than those related to food production. The review also identifies significant research gaps, including the limited number of studies on NGTs and the need for more comprehensive analyses of the social determinants of biotechnology acceptance. These findings provide a basis for future research and may support evidence-based communication and policymaking regarding agricultural biotechnology.

## 1 Introduction

Agriculture faces the challenge of ensuring food and nutrition security while simultaneously reducing its environmental impact. Sustainable agriculture aims to balance productivity with environmental protection, social wellbeing, and economic viability (Abdul Aziz et al., 2022; Tseng et al., 2020; Tripathi et al., 2022). Achieving these objectives has become increasingly difficult due to the pressures of climate change, resource scarcity, and growing food demand.

Recent advances in modern biotechnology are increasingly recognised as important tools for supporting the transition towards more sustainable and resilient agri-food systems. Accordingly, the European Commission (EC) has closely monitored these developments to evaluate how innovation can be harnessed while maintaining high safety standards. The development of genetically modified (GM) crops has enabled improvements in yield, pest and disease resistance, tolerance to environmental stresses, and nutritional quality (Kumar et al., 2020; Tyczewska et al., 2023). More recently, advances in genome editing have led to the development of new genomic techniques (NGTs), an umbrella term encompassing a range of modern methods that enable precise, targeted modifications of genetic material. Most of these techniques have been developed or have significantly advanced since 2001, when European Union (EU) legislation on genetically modified organisms (GMOs) was introduced (EC Study, 2021; Broothaerts et al., 2021; Graham et al., 2020; Kaur et al., 2022; Kardung et al., 2026; EFSA, 2026; EU legislation, 2026).

Despite their potential benefits, GMOs and, more recently, NGTs have raised environmental, health, ethical, socio-economic, and regulatory concerns (Eriksson et al., 2020; EC Study, 2021; Schiemann et al., 2019; Kjeldaas et al., 2023). Issues related to food and environmental safety, ethical considerations, governance transparency, and public trust in regulatory bodies have been particularly influential in shaping public perceptions in Europe, where the cultivation and commercialization of GM crops are subject to strict regulatory oversight (EC Study, 2021; Kjeldaas et al., 2023). Under current EU legislation, NGTs are regulated as GMOs, creating a barrier to the sustainable development of EU agriculture (CJEU - Court of Justice of the EU, 2018; Purnhagen et al., 2018; Ricroch et al., 2026). In 2023, the EC presented a draft proposal regarding plants obtained using certain NGTs, outlining detailed and specific provisions. The proposal applies solely to plants produced through site-directed mutagenesis and cisgenesis, as well as related food and feed products. Notably, the EC proposal excludes plants obtained through NGTs that introduce genetic material from a species that cannot be crossed (transgenesis), which remains governed by existing GMO legislation (EC, 2023). To better account for the different risk profiles of plants derived from NGTs, the proposal provides two marketing pathways for NGT plants. NGT plants that could also occur naturally or through conventional breeding will be verified based on predefined criteria specified in the application. NGT plants meeting these criteria will be treated as conventional crops, exempt from GMO regulation requirements. This means they will not require risk assessment and could be labelled similarly to conventional plants. Conversely, all other NGT plants will fall under current GMO legislation, necessitating a risk assessment and authorization process prior to market placement. For these plants, detection methods and monitoring requirements will be adjusted accordingly (EC, 2023).

Public perceptions play a crucial role in determining the societal acceptance of agricultural biotechnology. They influence not only the legitimacy of biotechnological innovations but also their adoption, market value, and commercial success. Attitudes towards GMOs and other biotechnological applications are shaped by several factors, including knowledge, awareness, perceived risks and benefits, trust in institutions, personal values, and broader societal trends (Woźniak et al., 2021; Woźniak-Gientka et al., 2022b). Therefore, the successful development and implementation of biotechnology depends not only on scientific progress and regulatory approval but also on public acceptance and engagement (Woźniak et al., 2021).

Within this context, the present study aims to synthesise the existing scientific evidence on public acceptance of agricultural biotechnology in Poland, focusing primarily on GMOs while also examining the emerging, though still limited, evidence regarding NGTs. Specifically, the review identifies patterns in public awareness, perceptions, and attitudes towards these technologies and analyzes the socio-demographic, cognitive, and contextual factors shaping their acceptance or rejection. Although the review focuses primarily on agricultural biotechnology, some studies on medical and industrial biotechnology are also included where relevant, providing insights into how application context affects public acceptance.

## 2 Social acceptance theoretical framework

Scientific and technological advancements, along with the services derived from them, can profoundly reshape societies and the lives of individuals; consequently, their deployment requires careful consideration of several factors, including social acceptance (Karasawa, 2025). In general terms, social acceptance of green transition technologies can be understood as “a favourable or positive response (including attitude, intention, behaviour and - where appropriate - use) relating to a proposed or *in situ* technology or socio-technical system by members of a given social unit (country or region, community or town and household, organization)” (Upham et al., 2015, p. 103). Although the concept is widely used in policy-oriented literature, it often lacks a precise definition. To improve conceptual clarity, Wüstenhagen et al. (2007) distinguished three dimensions of social acceptance: *socio-political acceptance*, referring to the support of technologies and policies by the general public, policymakers and key stakeholders; *community acceptance*, related to the approval of specific initiatives at the local level; and *market acceptance*, concerning the uptake of innovations by consumers, investors and firms. This framework provides a useful basis for analyzing how different actors engage with technological transitions. Although originally developed in the context of renewable energy transitions, the social acceptance framework has been increasingly applied to other sustainability-oriented innovations, including biotechnology and bioeconomy-related technologies, where similar challenges regarding trust, governance, stakeholder involvement, and public legitimacy occur.

Building on Wüstenhagen’s three-dimensional framework of social acceptance, Chin et al. (2014) categorized relevant stakeholders. For socio-political acceptance, stakeholders include the general public, policymakers, industry representatives, environmental organizations, and the media. Community acceptance involves farmers, residents, and local authorities, whereas market acceptance encompasses consumers and industry actors. Although the framework proposed by Wüstenhagen et al. (2007) helps differentiate between various dimensions of acceptance involving distinct groups of actors, its explanatory strength is limited by insufficient attention to the interactions between these dimensions across geographical levels, ranging from international and national to local contexts (Devine-Wright et al., 2017).

*Socio-political acceptance* is considered the broadest form of social acceptance, encompassing both political and technological dimensions. Wüstenhagen et al. (2007) noted that measurements from many countries indicate generally high socio-political acceptance of renewable energy technologies and associated policies. However, this overall positive perception has sometimes led to the assumption that social acceptance is unproblematic (Farstad et al., 2023). On the other hand, previous research has shown a lack of understanding of bioeconomy transitions (Zabaniotou and Kamaterou, 2019).

The second dimension of social acceptance of bioeconomy and biotechnology innovations is *community acceptance*, which describes the level of acceptance among local stakeholders - especially residents and local authorities - towards decisions related to the siting and development of bioeconomy projects (Wüstenhagen et al., 2007).

Social acceptance may also be reflected in the *market’s acceptance* of an innovation (Wüstenhagen et al., 2007). This form of acceptance can be examined within the innovation diffusion (Rogers, 2003), where the adoption of novel products by consumers is influenced by a dynamic communication process between individual adopters and their surrounding environment (Farstad et al., 2023; Devine-Wright et al., 2017). Therefore, market acceptance is critical not only from the perspective of consumers but also for investors, who influence the successful diffusion of innovations (Lundheim et al., 2022).

Within the broader concept of social acceptance, public perception describes how individuals interpret and evaluate emerging technologies and their potential implications. These evaluations are shaped not only by scientific knowledge but also by personal values, trust in relevant actors, previous experiences, and perceptions of potential risks and benefits. In contrast to scientific risk assessment, which focuses on the identification and evaluation of measurable hazards, risk perception reflects subjective interpretations shaped by cognitive, social, and cultural factors that influence attitudes towards agricultural biotechnology (Frewer et al., 2014; Eriksson et al., 2020; Kjeldaas et al., 2023). Research on agricultural biotechnology demonstrates that perceived risks and benefits are among the key determinants of public attitudes towards GMOs and other genetic technologies. Perceived benefits, such as enhanced agricultural productivity, improved resource-use efficiency, and environmental sustainability, are generally associated with more favourable attitudes. Conversely, concerns related to food safety, environmental impacts, ethical considerations, and the credibility of regulatory institutions tend to reduce public acceptance (Siegrist et al., 2000; Frewer et al., 2014). Furthermore, trust in scientists, regulatory authorities, and information sources plays a critical role in shaping opinions about technologies characterised by scientific complexity and uncertainty. Higher levels of institutional trust are generally associated with greater acceptance, whereas limited trust may strengthen scepticism and concerns regarding biotechnology applications (Frewer et al., 2014; Siegrist, 2008).

## 3 Literature search and analysis

This study provides a structured review of the literature on social acceptance of biotechnology in Poland. To identify relevant publications, searches were conducted in two bibliographic databases: Scopus and Web of Science. This search was conducted with the keywords from the following groups.

- Acceptance, opinion, perception;
- Biotechnology, genetically modified organisms/GMO (without human), new genomic techniques/NGTs;
- Poland.

In June 2026, searches were conducted in the Scopus and Web of Science databases using the article title, abstract, and keywords fields (Table 1, 2). One publication concerning new genomic techniques (NGTs) in Poland was identified in the Scopus or Web of Science databases. In this review, the terminology follows the terminology used in the original studies. The term biotechnology is used when referring to broader applications, whereas GMO/GM technologies refer specifically to genetically modified organisms and applications involving genetic modification. NGTs are treated as a distinct emerging category of genomic technologies.

**TABLE 1:** Criteria for searching and their results in the Scopus database.

Criteria for searching	Results – document found
Acceptance/perception/opinion, GMO/genetically modified organism, Poland	28
Acceptance/perception/opinion, biotechnology, Poland	28
All	56

Source: Authors’ compilation based on the reviewed literature.

**TABLE 2:** Criteria for searching and their results in the Web of Science database.

Criteria for searching	Results – document found
Acceptance of biotechnology in Poland; Biotechnology; Poland	6
Opinion on biotechnology in Poland; Biotechnology; Poland	9
Perception on biotechnology in Poland; Biotechnology; Poland	5
Acceptance of GMO in Poland; Or genetically modified organism; Poland	3
Opinion on GMO in Poland; Or genetically modified organism; Poland	1
Perception on GMO in Poland; Or genetically modified organism; Poland	1
All	25

Source: Authors’ compilation based on the reviewed literature.

The following inclusion criteria were applied during the selection of studies: (1) original articles written in English and Polish; (2) addressing issues of public perception and acceptance of biotechnology. Following the search, all publications were checked in terms of the type of article. Articles from different groups (GMO, genome editing, biotechnology, genetic engineering, GM food) were deleted in cases of duplicates to avoid repetition. Only original research (not review or opinion) has been chosen for further analyzis.

Scopus and Web of Science were chosen as the primary databases for the literature review due to their comprehensive coverage of peer-reviewed scientific publications and their widespread use in bibliometric and literature analyzis. Both databases index high-quality international journals and provide advanced search functionalities, enabling a comprehensive and reproducible search for relevant literature. Furthermore, their multidisciplinary scope ensures broad coverage of research areas related to biotechnology, GMOs and NGTs, which are central to this study.

The analyzis covers a 20 year period (2005–2025). A total of N = 81 papers were found. After removing 41 duplicate studies, 40 unique records remained. The remaining records were manually screened based on their titles and abstracts. Publications that did not meet the predefined inclusion criteria were excluded, resulting in the removal of 28 articles. Consequently, 12 original studies were selected for the final literature analyzis. The selected publications were analyzed qualitatively using thematic synthesis. The analyzis focused on research objectives, study characteristics, methodologies employed, and the main findings regarding public knowledge, perceptions, attitudes and acceptance of biotechnology, GMOs and NGTs in Poland.

## 4 Results

### 4.1 Characteristics of included studies

In Poland, empirical studies examining public perception and social acceptance of biotechnology remain relatively limited, particularly in the case of NGTs. The literature search identified 12 original studies examining public perception, awareness, and acceptance of biotechnology in Poland. The analyzed studies covered a period of 2 decades and included research conducted among different stakeholder groups, including the general public, consumers, students, farmers, and scientists. The studies differed substantially in terms of research objectives, methodologies, sample sizes, and analyzed applications of biotechnology.

Most identified studies focused on GMOs, particularly GM food, GM crops, and GM feed. Research specifically addressing NGTs remains very limited in Poland. Only one empirical study directly investigating public awareness and perceptions of NGTs was identified in the analyzed databases.

The included studies also varied regarding the analyzed dimensions of acceptance. Some studies focused on general knowledge and awareness of biotechnology, whereas others examined perceived risks and benefits, willingness to purchase or consume GM products, attitudes towards cultivation of GM crops, trust in institutions, or preferences regarding product labelling. A summary of the included studies is presented in Table 3.

**TABLE 3:** The main findings of the studies related to public acceptance of agricultural biotechnology.

No	Author(s) and year	Number of respondents	Target population	Purpose of the study	Methodology	Major themes
1	Mołodziejko et al. (2023)	194	Polish citizens (farmers, agricultural and food workers and professionals supporting agricultural production, as well as students of environmental sciences)	Analyse knowledge and attitudes towards NGTs	Survey, Pearson’s chi-square test	NGTs
2	Woźniak-Gientka et al. (2022b)	1008	Polish citizens over 15 years old	Analyse the awareness and opinion about genetic engineering, biotechnology and GM feed	Survey, Pearson’s chi-square test	Biotechnology, GMO
3	Nowakowska et al. (2021)	699	Polish citizens	Assess changes in public knowledge of modern genetic modifications in Poland and the level of social acceptance of GMO-containing products	Survey, Pearson’s chi-square test	GMO
4	Kubisz et al. (2021)	578	Students, consumers, producers, scientists	Describe the beliefs found about GMO foods	Survey	GMO, GM food
5	Pogodzińska (2020)	128	Farmers	Awareness of GMOs and the level of acceptance of the cultivation of GM crops	Survey	GMO
6	Hartmann et al. (2018)	1950	Population from United Kingdom, Sweden, Poland, France	Examine how consumers perceive “free-from” labels (e.g., gluten-, lactose-, and palm oil-free) in terms of product healthiness and willingness to pay a price premium, as well as to identify the psychological factors influencing these evaluations	Survey	GMO
7	Rzymski and Królczyk (2016)	1021	Polish citizens	Investigate the level of knowledge and the attitude towards the various uses of GMOs	Survey, Pearson’s chi square test	GMO
8	Jurkiewicz et al. (2014)	500	Adolescents completing secondary schools	Analyse opinion on GMO and GM food	Survey, Pearson’s chi square test	GMO, GM food
9	Małyska and Twardowski, 2014	250	Scientists, Scientific experts, farmers, Agricultural advisors	Examine attitudes toward various sectors of biotechnology	Survey, interviews, focus group	GMO
10	Zając et al. (2012)	745	Students of medical disciplines	Assess respondents’ knowledge of biotechnology, genetic engineering, and GMOs, as well as their willingness to trust and prefer GM food over conventional alternatives	Survey	GMO
11	Lubiatowska-Krysiak and Twardowski, 2008	30	Polish population	Attitudes of society towards GM food	Focus group interviews	GMO
12	Twardowski (2007)	1005	Polish population >15 years	Examine public opinion on biotechnology	Survey	Biotechnology, GM food

Source: Authors’ compilation based on the reviewed literature.

### 4.2 Knowledge and awareness of biotechnology, GMOs and NGTs in Poland

Studies conducted in recent years suggest that knowledge of biotechnology and genetic engineering among the Polish public remains at a relatively low level. Mołodziejko et al. (2023), who investigated awareness of NGTs among Polish respondents (N = 194), demonstrated that although more than half of participants had encountered the term “NGT”, only a small proportion were able to correctly define the concept. Nearly half of respondents had never heard about NGTs before. The study also revealed limited understanding of the legal status and market availability of NGT-derived products (Mołodziejko et al., 2023).

Comparable limitations in knowledge have been observed for GMOs. Rzymski and Królczyk (2016), analyzing public attitudes towards GMOs among Polish respondents (N = 1021), reported that less than half of participants were able to correctly define the term GMOs. A considerable proportion of respondents associated GMOs exclusively with food products or were unable to provide any explanation of the concept. Importantly, despite limited knowledge, respondents expressed interest in obtaining additional information. More than half of participants stated that their knowledge regarding GMOs was insufficient (Rzymski and Królczyk, 2016).

Similar findings were presented in the Report (2012), published by the Copernicus Science Centre, which found that many Poles declared limited familiarity with GMOs, as nearly half of respondents had never heard of the term (N = 1005). Three-quarters of respondents had difficulties in correctly interpreting the acronym GMO. Although nearly one-fifth associated GMOs with food, only 3.3% of responses reflected a scientifically correct understanding of the concept (Report, 2012). According to the Special Eurobarometer 341 (2010), the majority of Polish respondents declared that they had previously heard about GM food (N = 1000). Nevertheless, most respondents had never actively searched for information on this topic. Importantly, Nowakowska et al. (2021) reported a gradual increase in the level of public knowledge about GMOs in Poland in recent years (N = 699), while Woźniak-Gientka et al. (2022b) indicated that slightly more than half of Poles believed that products obtained through genetic engineering techniques, such as medicines, fruits and vegetables, and food additives, were available on the Polish market (N = 1008).

Research conducted among students also indicates that recognition of the term GMO does not necessarily translate into a comprehensive understanding of biotechnology. Zając et al. (2012) found that most students correctly identified the meaning of the GMO abbreviation and believed that GM food was already available on the Polish market (N = 745). Similarly, Jurkiewicz et al. (2014) reported that secondary school graduates demonstrated limited knowledge regarding the health effects of GMO consumption (N = 500).

### 4.3 Public attitudes and acceptance of different biotechnology applications

The reviewed literature consistently indicates that public acceptance of biotechnology in Poland is strongly dependent on the intended application of the technology. The analyzed studies demonstrate a clear distinction between agricultural and food-related applications, which are often associated with higher levels of concern, and non-food applications, particularly medical and environmental uses, which generally receive greater public support.

According to Mołodziejko et al. (2023), respondents most commonly associated potential NGT applications with healthcare, plant breeding, and the agri-food sector. Approximately 40% of respondents believed that products obtained using NGTs were already available within the EU and Poland, while awareness of the regulatory framework governing these technologies remained incomplete (Mołodziejko et al., 2023).

As reported by Copernicus Science Centre (2012), the majority of respondents expressed the view that the introduction of GMOs in agriculture primarily serves the interests of the companies producing these organisms and that GMO-based foods negatively affect consumer health (N = 1005). These perceptions highlight prevailing public concerns and scepticism regarding the motivations behind and safety of GMO applications in Poland. Moreover, Poles had insufficient knowledge regarding GMOs practical applications. Maize was the plant most frequently associated with genetic modification, followed by soybean and rapeseed. Maize is an important energy source in animal diets. At the same time, soybean, particularly soybean meal, is a major source of high-quality protein and essential amino acids, making both crops key components of livestock and poultry feed. At the same time, a large proportion of respondents were unaware that GMOs are used in the production of certain medicines, such as insulin (N = 382). These findings suggest that public understanding of biotechnology is often restricted to food-related applications, while broader industrial and medical uses remain less recognised. One in six Poles reported that they checked food products labels for the presence of GMO-based ingredients. A majority of Polish respondents (72%), however, perceived genetic modification as a method used to develop new plant varieties with improved performance in cultivation (Report, 2012).

Results of the Special Eurobarometer 341, 2010 found that public attitudes towards GM food were predominantly sceptical, as only a small proportion of respondents perceived GM food as beneficial for the Polish economy. Moreover, a substantial share of respondents questioned the safety of GM food for future generations and considered such food to be fundamentally unnatural (N = 1000).

Woźniak-Gientka et al. (2022b) demonstrated that public attitudes towards GM food in Poland remain predominantly sceptical. More than half of respondents supported banning the production and sale of transgenic food products. Opposition was also expressed towards the use of GM feed, as many respondents (62%) believed that products obtained from animals fed with GM feed could negatively affect human health. Over half of respondents supported restrictions on GM feed use even if this resulted in increased costs of milk, eggs, and poultry production. At the same time, only approximately one-third of respondents considered the cultivation of GM crops in Poland desirable in the context of food and feed security. According to Jurkiewicz et al. (2014) more than half of the respondents believed that food produced from GMOs poses a risk to human health.

Consumer willingness to purchase GM products also remains limited. Woźniak-Gientka et al. (2022b) showed that more than half of Polish respondents declared that they would not accept purchasing transgenic food products, regardless of their potential benefits. However, acceptance increased when specific consumer-oriented advantages were presented. Among the analyzed product characteristics, improved nutritional value generated the highest level of support, although only about one-third of respondents declared willingness to purchase such products.

Similarly, Rzymski and Królczyk (2016) found that approximately 60% of respondents opposed the production and distribution of GM food in Poland. The main concerns were related to potential health consequences, including cancer risks and allergic reactions, as well as possible negative environmental effects. These concerns demonstrate that perceived risks remain a dominant factor shaping attitudes towards agricultural biotechnology.

Research among farmers indicates that scepticism towards GM crops is also present among agricultural stakeholders. Pogodzińska (2020), analyzing attitudes among Polish farmers (N = 128), reported relatively low acceptance of GMO crops and GM food. Farmers frequently perceived GM technologies as primarily beneficial for large biotechnology companies rather than individual agricultural producers. Almost half of respondents were unable to assess whether GMO cultivation would be economically advantageous for their own farms, while only 24% considered GM crop cultivation economically beneficial.

At the same time, farmers recognised some potential advantages associated with agricultural biotechnology. These included increased yields, improved resistance to pests and diseases, and possible reductions in production costs. However, these perceived benefits were insufficient to overcome concerns regarding health, environmental impacts, and dependence on biotechnology companies. Pogodzińska (2020) found that nearly 64% of surveyed farmers opposed the import of GM soybeans and maize for feed production. In contrast, applications outside food production, such as biotechnology use in biofuel production and the paper industry, received higher acceptance levels. In another study, Kubisz et al. (2021) reported that students expressed more positive attitudes towards the potential benefits of GM technologies than consumers did, whereas farmers were the most sceptical group (N = 578).

Research conducted among scientists provides a different perspective. Małyska and Twardowski, 2014 found that scientists were generally more supportive of GM technologies than consumers or farmers, particularly regarding applications in industrial production, medicine, and pharmaceuticals. However, opinions concerning GM food and feed remained more divided, indicating that even among expert groups, acceptance depends strongly on the application context.

Overall, the evidence suggests that resistance towards biotechnology in Poland is particularly strong when genetic modification is directly associated with food consumption. Concerns appear to focus less on the technology itself and more on perceived consequences, including health risks, environmental uncertainty, and lack of trust in companies involved in biotechnology development.

In contrast to food applications, biotechnology used in medicine, pharmaceuticals, and environmental protection received substantially greater public acceptance. Woźniak-Gientka et al. (2022b) showed that the highest level of support concerned the use of microorganisms in environmental remediation and wastewater treatment. Similarly, public support for biotechnology research related to vaccines and medicines was considerably higher than for agricultural applications. Comparable trends were identified by Małyska and Twardowski, 2014, who reported that scientists strongly supported the use of GMOs in medicine, pharmaceutical production, and industrial applications, while opinions regarding food and feed applications were considerably more divided.

### 4.4 Factors shaping acceptance: perceived risks and benefits, trust, socio-demographic differences

Although knowledge and awareness influence attitudes towards biotechnology, they do not fully determine acceptance. Perceptions of risks and benefits, trust in information sources and regulatory institutions, as well as socio-demographic characteristics and social group membership, contribute to the complexity of public attitudes towards biotechnology.

Rzymski and Królczyk (2016) showed that opposition towards GM food was primarily associated with concerns regarding potential health consequences, including cancer risks and allergic reactions, as well as possible negative environmental effects. Similarly, Nowakowska et al. (2021) reported that more than half of respondents expressed concerns about potential health risks associated with the consumption of GMO-containing food products (N = 699). These findings indicate that risk perceptions remain a central element shaping public attitudes towards agricultural biotechnology.

Zając et al. (2012) found that concerns regarding potential risks to human health and the natural environment were widespread (N = 745). The findings indicated that respondents were more likely to associate GM food with potential benefits than with risks. The most frequently perceived benefits included increased resistance to pests and the belief that GMOs do not adversely affect the natural environment. Nevertheless, about 45% of respondents believed that GM food may contain pathogenic factors, indicating the persistence of food safety concerns. By contrast, relatively few respondents considered GMOs to pose a direct risk to human health or the environment (Zając et al., 2012). Jurkiewicz et al. (2014) reported that students recognised some potential agricultural benefits, including increased yields, improved disease resistance, and reduced pesticide use (N = 500). With regard to environmental concerns, respondents most frequently pointed to the replacement of conventional plant varieties, biodiversity loss through gene flow, the emergence of herbicide-resistant weeds, and the uncontrolled spread of GM plants.

Kubisz et al. (2021) noted that among the perceived advantages of GM technologies, respondents most frequently cited higher crop yields, improved pathogen resistance, and increased tolerance to drought (N = 578). In contrast, lower levels of support were observed for more indirect or global benefits, such as the potential contribution of GM technologies to reducing world hunger or their application in medicine production. Among non-food applications, the production of biofuels received the highest level of acceptance. The findings suggest that public concerns are directed less towards genetic modification as a technology itself and more towards its perceived consequences, particularly in the context of food production and potential risks to human health (Kubisz et al., 2021).

Qualitative research conducted by Lubiatowska-Krysiak and Twardowski, 2008 demonstrated that consumers often perceived information about GMOs as unreliable and influenced by commercial interests. Biotechnology companies were frequently associated with profit-oriented motivations, which reduced public confidence in the benefits and safety of GM products. These findings highlight that acceptance depends not only on scientific evidence but also on the perceived credibility of actors communicating information about biotechnology.

Scientists and educational institutions were generally identified as more trusted sources of information. Rzymski and Królczyk (2016) reported that respondents considered scientists and teachers among the most reliable sources. This suggests that science communication may play an important role in reducing information gaps and supporting more informed public discussions. Jurkiewicz et al. (2014) found a generally low level of trust in GM-related information and products. While 64% of respondents regarded media reports on the benefits of GM crops as untrustworthy and unreliable, nearly 70% of the adolescents surveyed also reported a lack of trust in food produced from GMOs.

Regulation and transparency were also identified as important components of public acceptance. Several studies showed strong public support for governmental control, mandatory labelling, and regulatory oversight of genetically modified products. Woźniak-Gientka et al. (2022b) reported that approximately three-quarters of Polish respondents believed that research and applications involving biotechnology and genetic engineering should be controlled and regulated by public authorities. Similarly, Rzymski and Królczyk (2016) found strong support for mandatory GMO labelling, with many respondents expecting detailed information regarding the presence of genetically modified ingredients. Zając et al. (2012) reported that most students believed that GM food products should be labelled, whereas Twardowski (2007) found that the vast majority of respondents considered state administrative supervision of biotechnology to be necessary.

However, the relationship between information availability and consumer behaviour appears complex. Although respondents frequently supported GMO labelling, actual purchasing decisions were not always consistent with declared attitudes. Nowakowska et al. (2021) found that nearly 54% of respondents did not consider GMO information on product labels when making purchasing decisions, indicating a gap between stated preferences and everyday behaviour. Cross-country research also suggests that Polish consumers place considerable importance on transparency and product information. Hartmann et al. (2018) found that respondents from Poland were more likely than participants from Sweden and the United Kingdom to perceive GMO-free labelled products as healthier. Polish consumers also demonstrated a relatively high willingness to pay more for products labelled as GMO-free, highlighting the importance of labelling and perceived safety in shaping market acceptance.

The reviewed literature indicates that public acceptance of agricultural biotechnology in Poland also varies according to socio-demographic characteristics and social group membership. Factors such as gender, age, education, and professional background influence perceptions of biotechnology, although their effects are not always consistent across studies. Gender differences have been observed particularly in relation to risk perception. Nowakowska et al. (2021) found that women were more likely than men to express uncertainty regarding the use of NGT-derived products. Approximately 56% of female respondents reported having no clear opinion on NGT products, whereas 39% of male respondents declared willingness to use such products. Similarly, Zając et al. (2012) reported that women more frequently associated GM food with potential risks to human health, whereas men more often emphasised possible agricultural benefits, such as pest resistance. Age has also been identified as a relevant factor influencing attitudes towards agricultural biotechnology. Nowakowska et al. (2021) showed that younger respondents were generally more supportive of GM crop cultivation than older age groups. Among respondents aged 20–35 years, 61% expressed favourable attitudes towards GM crop cultivation, compared with only 14% among respondents aged 60 years and older. Older respondents were also more likely to perceive products derived from animals fed with GM feed as potentially harmful.

The relationship between education and acceptance appears more complex. While higher education is sometimes associated with greater willingness to accept new biotechnologies, other studies indicate that more educated groups may also demonstrate increased critical reflection and awareness of potential risks. Woźniak-Gientka et al. (2022b) found only minor differences between education groups regarding support for transgenic food production, whereas Nowakowska et al. (2021) reported that respondents with higher education levels were more willing to accept and use NGT-derived products compared with those with secondary education. According to the Copernicus Science Centre (2012), respondents who declared greater familiarity with the topic tended to be younger and better educated (Report, 2012).

Differences between stakeholder groups are particularly important for understanding acceptance of agricultural biotechnology. Kubisz et al. (2021) demonstrated that students expressed more positive attitudes towards the potential benefits of GM technologies than consumers, whereas farmers represented the most sceptical group. Farmers’ attitudes may reflect their direct involvement in agricultural production and concerns regarding economic dependency, market conditions, and potential consequences for farm management. Małyska and Twardowski, 2014 reported generally favourable attitudes toward GM technologies among scientists.

## 5 Discussion and conclusion

The development of agricultural biotechnology, including NGTs, is increasingly discussed in the context of the transition towards more sustainable agricultural systems. However, the development and regulatory governance of these technologies are not solely matters of scientific and technical progress. Public perceptions, societal attitudes, and trust in science, institutions, and regulatory processes may also shape how these technologies are perceived and received. This social dimension is particularly relevant in the European context, where biotechnology governance extends beyond scientific risk assessment to encompass ethical, social, and political considerations (Eriksson et al., 2020; Ricroch et al., 2026; Kjeldaas et al., 2023).

The regulatory environment plays an important role in shaping societal attitudes toward biotechnology and NGTs. In recent years, these technologies are increasingly considered a promising approach to addressing major global challenges, including climate change adaptation, food security, and the sustainability objectives of the (European Green Deal, 2019; Ricroch et al., 2026; Pokrivcak et al., 2026; Molitorisová et al., 2025; Purnhagen et al., 2018; Purnhagen et al., 2023). The ongoing revision of the European regulatory framework reflects attempts to better align legislation with scientific progress and the distinct characteristics of genome-edited plants (EC, 2023). Nevertheless, changes in legislation alone are unlikely to increase societal acceptance. Previous studies demonstrate that public confidence depends not only on regulatory oversight but also on the transparency, credibility and perceived legitimacy of governance processes (Strobbe et al., 2023; Eriksson et al., 2020).

Beyond the regulatory framework, perceptions of the benefits, risks, and applications of biotechnology may shape how different groups respond to NGT-derived products (Pokrivcak et al., 2026; Bearth et al., 2024; Qaim, 2020). Studies indicate that individuals who are more familiar with biotechnology are generally more likely to perceive such technologies as safe and beneficial. However, research also showed that knowledge alone does not fully determine acceptance, as attitudes are simultaneously shaped by social values and perceptions of technological risks (McFadden et al., 2024). Although experts recognize numerous benefits of genetic modification, this technology remains widely unaccepted and may even be rejected by end-users (Sijtsema et al., 2016). NGTs can support the agriculture and food sectors in various ways, benefiting different stakeholders across the food chain. Farmers would benefit from the increased availability of crops adapted to the sector’s needs, such as climate change resilience, pest resistance, increased yields, and reduced reliance on fertilizers and pesticides. Consumers would have a wider selection of food products with improved taste, better nutritional properties, or reduced amounts of allergenic substances while supporting sustainability. It is important to note that consumers will choose which products to buy from those available on the market. Producers and traders may also experience advantages such as reduced use of natural resources and emissions associated with food transportation, as well as improved processing properties (EC, 2025). At the same time, the availability of such benefits does not necessarily translate into positive public responses. This highlights the importance of understanding how different stakeholders perceive the potential benefits and risks associated with NGTs and how these perceptions may influence their attitudes towards their development and use.

In the EU over the past 2 decades, public opinion research has shown a gradual but measurable shift in attitudes toward biotechnology (Woźniak-Gientka et al., 2022a). Woźniak et al. (2021) reported that European societies increasingly recognize the medical benefits of biotechnology in preventing and treating diseases as well as reducing disabilities, while genetically modified (GM) products - particularly GM plants - remain less widely accepted. Other surveys suggested a gradual decline in the proportion of Europeans expressing reluctance towards GM food, from 86% in 1999 to 66% in 2010 and 60% in 2019 (Sikora and Rzymski, 2021). Public attitudes towards agricultural biotechnology are also not uniform across countries or applications, suggesting that perceptions are shaped by the specific characteristics and perceived benefits and risks of individual technologies.

The body of empirical research on public acceptance and opinion of biotechnology in Poland remains fragmented and has historically focused predominantly on GMOs, particularly GM food, crops, and feed. The identified studies also vary considerably in terms of research objectives, target populations, sampling approaches, and methodological designs, which limits the comparability of their findings. In contrast, empirical research specifically addressing public awareness and perceptions of NGTs remains extremely limited, with only one study identified in the analyzed databases. Moreover, the relatively small sample size (N = 194) and limited generalisability of the available evidence further constrain the extent to which conclusions about public acceptance of NGTs can be drawn. Overall, the current body of evidence remains insufficient to provide a robust and comprehensive assessment of public acceptance of NGTs in Poland.

Despite this methodological heterogeneity, several recurring patterns emerge across the studies addressing public perceptions of GMOs. First, although the awareness of biotechnology-related terms is relatively widespread, detailed knowledge and a clear understanding of biotech concepts remain limited. Research conducted over the past 2 decades indicates a persistent gap between self-reported familiarity and factual knowledge, as a significant proportion of respondents cannot correctly define key terms or articulate the scope of biotechnology applications. This suggests that public understanding is largely superficial and based on terminology recognition rather than substantive knowledge. This pattern is not unique to Poland. European studies similarly indicate a substantial gap between awareness of biotechnology-related terms and actual understanding of genome editing and its applications. For example, recent studies from the Czech Republic and Germany reported limited public familiarity with genome editing and substantial uncertainty regarding its applications and regulatory status (Čadová et al., 2025; Kato-Nitta et al., 2023). Similarly, Busch et al. (2022), based on data from Austria, Germany, Italy, Canada and the United States, found that although genetic modification was relatively widely known, self-assessed knowledge of genome editing remained low. Nordic countries, including Finland, Sweden, Denmark and Norway, stand out in terms of attitudes and perception of GM food, GMO, and genome editing (Woźniak-Gientka et al., 2022a; Eriksson et al., 2018). As noted by Eriksson et al. (2018), this may result from the early adoption of plant gene technologies in this region. Thus, the limited understanding of NGTs observed in Poland appears to reflect a broader challenge in communicating emerging genome-editing technologies rather than a country-specific phenomenon.

Second, there is a clear asymmetry in acceptance across biotechnology application domains. Medical and pharmaceutical applications are more socially acceptable compared to applications in food production, agriculture, and animal feed. This pattern suggests that public attitudes are not directed toward biotechnology *per se* but are strongly context-dependent and shaped by the technology’s perceived purpose. Food-related applications, in particular, elicit stronger resistance, reflecting increased sensitivity to perceived risks. The application-specific nature of biotechnology acceptance observed in Poland is also consistent with findings reported in Sweden, Switzerland, and Italy. Recent research suggests that attitudes toward NGTs are not uniformly positive or negative but vary according to the intended purpose and perceived benefits of a particular application. For example, the Swedish public expressed relatively high acceptance of gene editing when applied to crops addressing food security or specific agricultural problems, while studies in Switzerland, the United States and Italy similarly found differentiated attitudes toward particular gene-edited food products (The Swedish Report, 2025; Bearth et al., 2024; Morandi et al., 2025). These findings are comparable with the Polish evidence, as they likewise indicate that acceptance depends on the intended application rather than on the technology itself.

Third, risk perceptions dominate the public debate on biotechnology. Concerns about human health, environmental safety, and the “unnaturalness” of GM products are emphasized in Polish research. At the same time, perceived benefits, such as increased agricultural productivity, improved resistance to stressors, and contributions to medical innovation, are acknowledged but appear to have less influence on shaping general attitudes. This imbalance suggests that risk perceptions play a more decisive role than benefit perceptions in shaping public opinion. European studies reveal substantial variation in the perceived safety of GM foods, both across countries and between population groups. While a majority of Montenegro residents perceived GMOs as posing risks to human and animal health, more than half of the Italian general public and the majority of Italian scientists considered GM foods safe to eat (Velimirović et al., 2026; Pappalardo et al., 2021). According to Romeo Lironcurti et al. (2024), four in ten Italians would buy a product made from GM crops. As reported by Morandi et al. (2025), Italian consumers (N = 1045) were willing to purchase wine produced from NGTs. Notably, 59% of respondents indicated a positive response, whereas 19% said that they would not buy it. In the UK, a significant proportion of respondents considered gene editing to be excessively risky, and uncertainty about its safety remains prevalent among consumers (van Mill et al., 2018). This variation is consistent with the Polish findings, which also indicate that risk perceptions differ depending on the application and perceived consequences of biotechnology.

Finally, the reviewed literature suggests that transparency, labelling, and institutional oversight are important elements shaping public perceptions of agricultural biotechnology in Poland. The discrepancy between strong support for mandatory labelling and the limited attention paid to GMO information during actual purchasing decisions indicates that labelling alone may not substantially change consumer behaviour. Rather, its importance may lie in providing transparency, reinforcing consumers’ perceived control over food choices, and signalling product safety. Similar findings have been observed in Czech Republic and Montenegro, although the importance attached to food labelling varies across populations. In the Czech Republic there is strong support for the clear labelling of products containing ingredients derived from genetically engineered crops, while a substantial proportion of respondents remains sceptical about their safety (Čadová et al., 2025). In Montenegro, by contrast, a considerable proportion of consumers reported not paying attention to GMO labelling, suggesting that the availability of information does not necessarily translate into active use of such information in purchasing decisions (Velimirović et al., 2026).

This review is subject to certain limitations, primarily related to the heterogeneity of the reviewed studies and the limited body of empirical evidence regarding NGTs in Poland. The reviewed studies differ significantly in terms of research objectives, sample characteristics, sampling methods, and measurement approaches, which limits the ability to directly compare the results obtained. Furthermore, only one study concerning the public perceptions of NGTs in Poland was identified in the literature reviewed. Consequently, the available data do not yet allow for the formulation of reliable conclusions regarding the scale and determinants of public acceptance of NGTs in Poland.

The reviewed literature highlights substantial knowledge gaps among the Polish public regarding both GMOs and NGTs. Although awareness of biotechnology has increased over time, understanding of its specific applications and regulatory context remains limited. In particular, NGTs are still poorly understood, with many respondents unable to correctly define the concept or distinguish between products available within and outside the EU. Importantly, increased awareness does not necessarily translate into higher acceptance, suggesting that attitudes are shaped not only by knowledge, but also by underlying values, trust, and perceived control over the technology. In this context, transparent and evidence-based communication with the public is essential. Effective dialogue among scientists, policymakers, industry representatives, and consumers may support informed public debate and help minimise conflicting narratives surrounding NGT applications. Future research should prioritize longitudinal and theory-driven studies to better examine how knowledge, trust, and value orientations shape public attitudes towards biotechnology in Poland.

## Glossary

Genetic engineering: Term commonly used in the United States. The United States Department of Agriculture (USDA) defines genetic engineering as the “manipulation of an organism’s genes by introducing, eliminating or rearranging specific genes using the methods of modern molecular biology, particularly those techniques referred to as recombinant DNA techniques.”
Genetically modified organism (GMO): Term used in the European Union legislation, and defined in Directive 2001/18/EC, as “an organism, except for human beings, in which the genetic material has been altered in a way that does not occur naturally by mating and/or natural recombination.”
New genomic techniques (NGTs): NGTs, also called precision breeding, refer to the techniques developed since the adoption of the EU’s legislation on genetically modified organisms (GMO) in 2001. NGTs are breeding techniques that alter the genetic material of plants, animals or microorganisms.
Targeted mutagenesis: A technique that induces specific mutation(s) in targeted locations of the genome without inserting new genetic material.
Cisgenesis: An approach that modifies the genetic material of an organism with a sequence from a donor from the same species or one closely related.
